# The Impact of Adding Digital Breast Tomosynthesis to BI-RADS Categorization of Mammographically Equivocal Breast Lesions

**DOI:** 10.3390/diagnostics13081423

**Published:** 2023-04-15

**Authors:** Rania Mostafa Hassan, Yassir Edrees Almalki, Mohammad Abd Alkhalik Basha, Sharifa Khalid Alduraibi, Mervat Aboualkheir, Ziyad A. Almushayti, Asim S. Aldhilan, Sameh Abdelaziz Aly, Asmaa A. Alshamy

**Affiliations:** 1 Department of Diagnostic Radiology, Faculty of Human Medicine, Zagazig University, Zagazig 44519, Egypt; raniahassan@medicine.zu.edu.eg (R.M.H.); mohammad_basha76@yahoo.com (M.A.A.B.); aashami@medicine.zu.edu.eg (A.A.A.); 2 Division of Radiology, Department of Internal Medicine, Medical College, Najran University, Najran 61441, Saudi Arabia; 3 Department of Radiology, College of Medicine, Qassim University, Buraidah 52571, Saudi Arabiaa.aldhilan@qu.edu.sa (A.S.A.); 4 Department of Radiology and Medical Imaging, College of Medicine, Taibah University, Madinah 42353, Saudi Arabia; maboualkheir@taibahu.edu.sa; 5 Department of Diagnostic Radiology, Faculty of Human Medicine, Benha University, Benha 13511, Egypt; drsamehaly75@gmail.com

**Keywords:** digital mammography, tomosynthesis, equivocal breast lesions

## Abstract

Digital mammography (DM) is the cornerstone of breast cancer detection. Digital breast tomosynthesis (DBT) is an advanced imaging technique used for diagnosing and screening breast lesions, particularly in dense breasts. This study aimed to evaluate the impact of combining DBT with DM on the BI-RADS categorization of equivocal breast lesions. We prospectively evaluated 148 females with equivocal BI-RADS breast lesions (BI-RADS 0, 3, and 4) with DM. All patients underwent DBT. Two experienced radiologists analyzed the lesions. They then assigned a BI-RADS category for each lesion according to the BI-RADS 2013 lexicon, using DM, DBT, and integrated DM and DBT. We compared the results based on major radiological characteristics, BI-RADS classification, and diagnostic accuracy, using the histopathological examination of the lesions as a reference standard. The total number of lesions was 178 on DBT and 159 on DM. Nineteen lesions were discovered using DBT and were missed by DM. The final diagnoses of 178 lesions were malignant (41.6%) and benign (58.4%). Compared to DM, DBT produced 34.8% downgrading and 32% upgrading of breast lesions. Compared with DM, DBT decreased the number of BI-RADS 4 and 3. All the upgraded BI-RADS 4 lesions were confirmed to be malignant. The combination of DM and DBT improves the diagnostic accuracy of BI-RADS for evaluating and characterizing mammographic equivocal breast lesions and allows for proper BI-RADS categorization.

## 1. Introduction

Breast cancer is the most common cancer among females all over the world, accounting for approximately 27% of all cancer cases [1]. Early discovery has become a key task in reducing morbidity and mortality rates in women with breast cancer [2]. A breast mass is a common mammography finding, defined as a two-view lesion with partially or completely convex boundaries. Other non-mass findings can also be observed, including global, focal, or developing asymmetry, microcalcification, and architectural distortion [3]. Strategies to detect them by screening mammograms include a symmetrical comparison of breasts, comparison with previous mammograms, identification of parenchymal contour distortions, and observation of retromammary fat and associated findings [4]. Awareness of these findings is essential for effective diagnosis and treatment. Missing or misinterpretation may be due to limitations in diagnostic tools, radiologist exhaustion during the screening program, or indistinct tumor characteristics on the mammogram [5]. Accurate diagnosis is crucial for proper treatment and prognosis [6].

The main breast imaging method for the early recognition and diagnosis of breast malignancies is digital mammography (DM), but there are still some limitations [7]. Dense breasts are one of the main restrictions in DM [8]. Owing to the diminished contrast between breast tumors and surrounding breast tissues, DM has low specificity and sensitivity in females with radio-dense breasts, and the sum of the parenchyma might mask the lesions [9]. Digital breast tomosynthesis (DBT) can overcome this obstacle by eliminating or reducing the tissue overlap. DBT is an alteration of the DM unit to acquire thin sections and three-dimensional (3D) volume data [8]. The role of DBT in the exclusion of suspicious abnormalities found during screening is considered a crucial diagnostic function [10]. DBT also allows the visualization of masses that are not obvious in DM [11]. A clearer description of DBT should make it easier to distinguish between benign and malignant lesions [8]. Although the radiation dose of two-dimensional (2D) mammography and tomosynthesis exceeds that of 2D mammography alone, the combined dose of 2D mammography and DBT is still below the Food and Drug Administration (FDA) safety limit of 3 mGy per radiation dose used for screen-film mammography [12].

The Breast Imaging Reporting and Data System (BI-RADS) was originally established to enable radiologists to disclose their concerns that DM may miss breast lesions due to high breast density; however, it has been broadly used in research on breast cancer and DM performance [13,14]. Many previous studies have reported that adding DBT to screening procedures and diagnostic environments has many benefits [15,16,17]. The DM findings can be seen more clearly in tomographic images. Moreover, DBT can upgrade malignant lesions in BI-RADS that are falsely judged by DM [18], better diagnose BI-RADS 0 findings in dense breasts [19], and characterize uncertain lesions determined by DM (BI-RADS 3 and 4) [20]. Therefore, we conducted this prospective study to assess the impact of combining DBT with DM on BI-RADS classification of mammographically equivocal breast lesions (BI-RADS 4, 3, and 0) after using DM as the first imaging modality. In addition, we compared DM and DBT.

## 2. Patients and Methods

### 2.1. Ethical Consent

The study was approved by the institutional review board (approval No: ZU-IRB# 9202; approved 12 January 2021). Written informed consent was obtained from each patient for participation in the study. This study was conducted in accordance with the Code of Ethics of the World Medical Association (Declaration of Helsinki) for studies involving humans.

### 2.2. Population and Study Design

This prospective study was conducted from September 2021 to January 2023. The authors examined the radiology data system for patients categorized as BI-RADS 4, 3, and 0 on DM to be enrolled in the research process. They recorded the demographic, clinical, and mammographic imaging data of all the patients during the study period. The inclusion criteria were female ≥ 30 years, equivocal breast lesions by DM (BI-RADS 4 and 3), and dense breasts with symptoms (BI-RADS 0). Once enrolled, all patients were asked for another visit to undergo a DBT examination.

### 2.3. The Technique of DM and DBT

DBT examinations were performed within 14 days of DM examinations. The examinations were conducted on a full-field DM machine with 3D DBT (Senographe Essential GE Healthcare, Chicago, IL, USA). Each breast was pressed and carefully adjusted. Both procedures used two views for each breast: the mediolateral oblique (MLO) and the craniocaudal (CC). The 3D DBT acquired nine projections at a scan angle of 25°. The 3D volume of the compressed breast was reconstructed from 2D projections in a sequence of cuts (pictures) over the entire breast. For reading, images from both techniques were transferred to liquid-crystal display (LCD) displays. No more views were required since further editing, such as altering contrast, zooming, darkness, brightness, and inverting the background, could be performed while viewing the digital images on workstation LCD screens to aid in the detection of the lesion.

### 2.4. Interpretation and Data Analysis

DBT and DM images were transferred to the workstation for evaluation. The DM and DBT images were separated for review. Two experienced radiologists with more than 10 years of experience in breast imaging performed a comprehensive review of all images in consensus. The review was performed in three settings separated by one month to diminish the memory bias of the reviewers. In the first setting, two radiologists reviewed the DM images. After one month, the same two radiologists reviewed the DBT images. After another month, the same two radiologists reviewed the DM and DBT images together. Radiologists were unaware of clinical information and pathological reports. For each lesion, the following features on DM and DBT were individually assessed: density of the breast, lesion site, size, type of lesion (focal asymmetry or mass), mass features (density, margin, and shape), asymmetry (global, focal, simple, or developing), calcifications (distribution and morphology), and any other suspicious abnormalities. Three BI-RADS categories were assigned to each lesion (one for DM, one for DBT, and one for integrated DBT and DM). In the integrated protocol, we allocated a BI-RADS category based on the integrated features from each modality. A feature was considered positive when seen in at least one DM or DBT. When a finding was detected in both DM and DBT, it was considered positive. The major radiological characteristics, the BI-RADS categorization, and the diagnostic accuracy of the DM and DBT results were compared. 

### 2.5. Reference Standard

The final diagnosis was determined based on histopathological results after ultrasound-guided biopsy (*n* = 110), surgical mastectomy (*n* = 23), and stereotactic biopsy (*n* = 15). Two experienced pathologists examined the specimens and the definitive results were obtained by consensus. Biopsies were performed to ascertain the type of lesion at the request of the referring clinician.

### 2.6. Statistical Analysis

Statistical analysis was performed using SPSS software version 25 (IBM, 2017). Tables and figures were used to present the data. Means and standard deviations were used to represent continuous data. Frequencies and proportions were used to convey qualitative data. Qualitative data were analyzed using Pearson’s chi-square test. The McNemar test was used to evaluate the paired qualitative data. A lesion-based analysis was used to assess the diagnostic accuracy of DM, DBT, and the combination of DBT and DM. To determine the areas under the curve (AUCs), the receiver operating characteristic (ROC) curve analysis was used to determine the area under the curve (AUC). A statistically significant *p*-value of 0.05 was accepted.

## 3. Results

### 3.1. Research Sample

**Figure 1:**
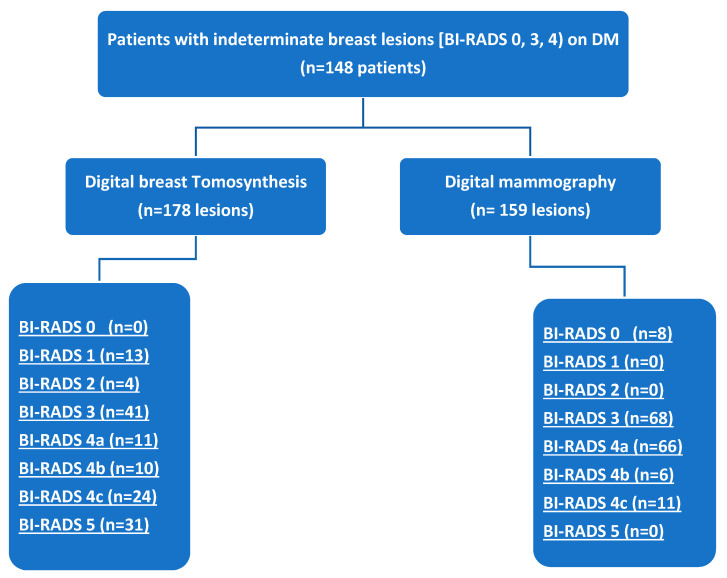
Flow chart of the study.

**Figure 2:**
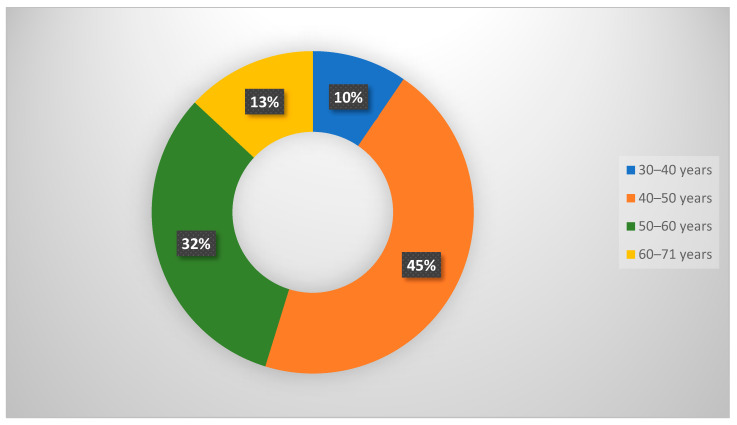
A pie chart shows the percentage of the age group distribution among the studied patients.

One hundred forty-eight female patients (mean age = 44.2 ± 7.3 years, range = 20–71 years) were enrolled. The patient data are summarized in Table 1. Every patient showed at least one breast lesion on DM, classified as BI-RADS 0, 3, or 4. Based on the current ACR lexicon of breast tissue density, our patients were in one of four groups: seven with ACR density A, 50 with ACR density B, 78 with ACR density C and 13 with ACR density D. On DM, we discovered 159 lesions and on DBT, 178 lesions were detected (Figure 1). Among the 178 lesions, 58.4% were benign and 41.6% were malignant. Fibrocystic disease was the most common benign disease (41.3%), whereas invasive ductal carcinoma was the most common malignant lesion (70.3%). The most commonly affected age group was 40–50 years (45%) (Figure 2).

### 3.2. DBT and DM Results

The DBT and DM results are summarized in Table 2. On DM, 85 lesions were non-mass and 74 were mass; on DBT, 38 lesions were non-mass and 140 were mass. Five of the 13 patients with architectural abnormalities and 5 of the 9 patients with microcalcifications on DM exhibited masses underneath DBT. DBT revealed overlapping normal glandular tissue in 13 patients, which was misinterpreted as a mass on DM. DBT detected 19 lesions that were missed on DM. We found that dense breasts (density C and D) (*n* = 16) displayed more of these lesions than non-dense breasts (density A and B) (*n* = 3).

### 3.3. Assignment of BI-RADS Categories by DBT and DM 

The categorization of BI-RADS lesions according to DM is presented in Table 3.

Table 4 shows the differences in the individual breast lesions between DBT and DM. In comparison to DM, DBT resulted in a 32% increase in BI-RADS categories (4.5% in BI-RADS 0, 7.3% in BI-RADS 3, 15.7% in BI-RADS 4a, 3.4% in BI-RADS 4b, and 1.1% in BI-RADS 4c) and a 34.8% decrease in BI-RADS categories (18.5% in BI-RADS 3 and 16.3% in BI-RADS 4a). Three downgraded BI-RADS 4 lesions were malignant, and all upgraded BI-RADS 4 lesions were malignant. DBT minimized the number of BI-RADS 3 and 4 lesions compared with DM (41 and 45 vs. 68 and 83, respectively).

### 3.4. Diagnostic Accuracy of DM, DBT and Integrated DBT and DM

The diagnostic accuracies of BI-RADS with integrated DBT and DM, BI-RADS with DBT, and BI-RADS with DM for detecting breast malignancies are presented in Table 5. We found that BI-RADS 4 and 5 were more predictive of malignant breast lesions and outperformed BI-RADS 5 alone in terms of diagnostic accuracy. Based on the BI-RADS version 2013, DBT demonstrated significantly higher accuracy, specificity, and sensitivity than DM in detecting breast cancer (*p* < 0.001). Compared to DM or DBT alone, the integration of DBT and DM considerably improved the accuracy of BI-RADS in the definition of malignant breast lesions (*p* < 0.001). 

### 3.5. Analyses of the ROC

**Figure 3:**
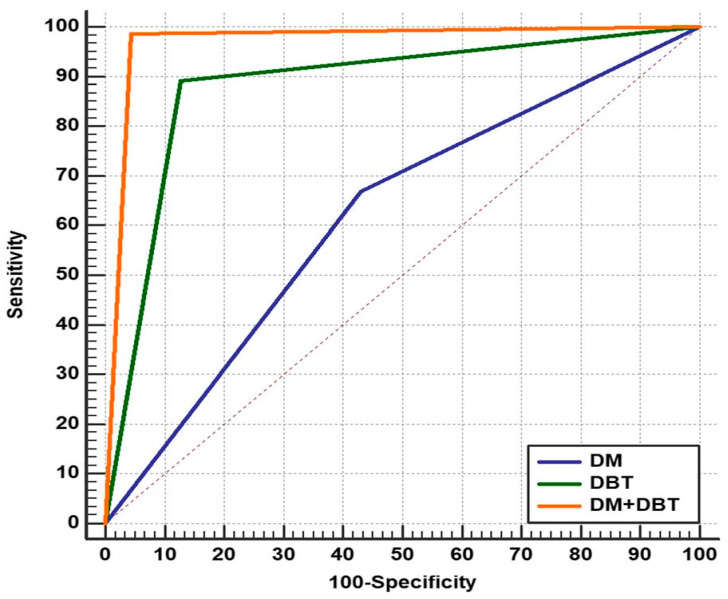
Comparison of the three ROC curves representing BI-RADS by DM, BI-RADS by DBT, and BI-RADS by integrated DBT and DM for the diagnosis of different breast lesions, as confirmed by histopathological examination as the gold standard.

Using the ROC curve (Figure 3), when the AUCs were compared, we observed that BI-RADS with DBT significantly outperformed BI-RADS with DM in breast cancer diagnosis (0.883 vs. 0.619, *p* = 0.0001) and BI-RADS with integrated DM and DBT significantly outperformed both BI-RADS with DM alone and BI-RADS with DBT alone (0.971, *p* = 0.0001).

**Figure 4:**
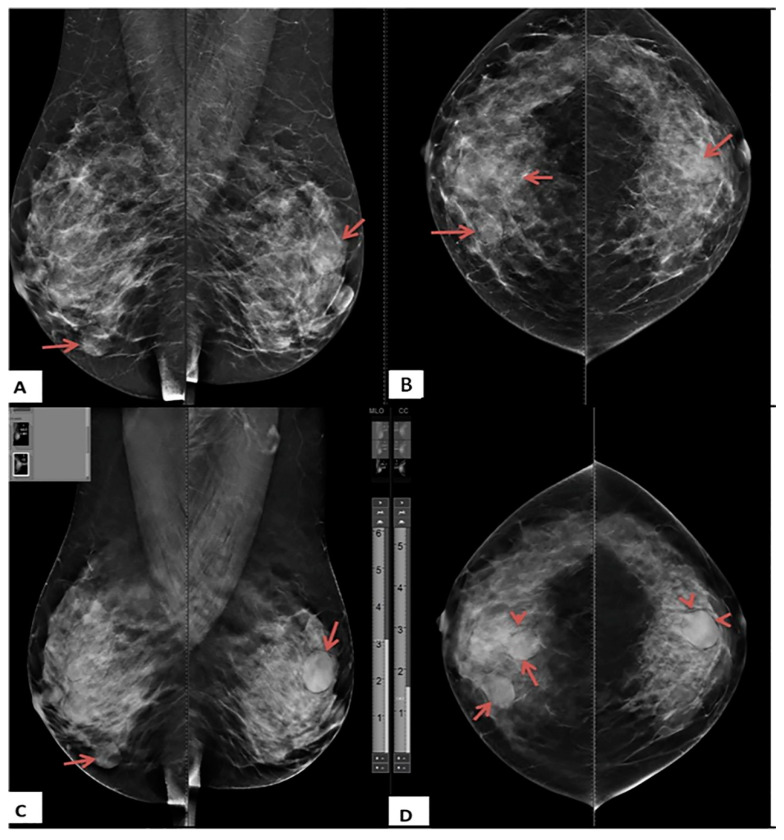
A 40-year-old female presented with bilateral breast lumps. (**A**) Mediolateral oblique and (**B**) Craniocaudal DM images of both breasts show heterogeneous dense breasts (ACR C) with left breast retroareolar, right breast retroareolar, and right breast lower inner quadrant dense lesions with obscured margins (arrows). No microcalcifications or speculated masses. (**C**) Mediolateral oblique and (**D**) Craniocaudal DBT images show more margin characterizations of the lesions, which are medium-dense, well-defined rounded lesions with smooth margins and a characteristic halo sign (arrowheads). The lesions were classified as BI-RADS 3 according to DM and BI-RADS 2 according to DBT. Histopathological examination revealed simple cysts.

**Figure 5:**
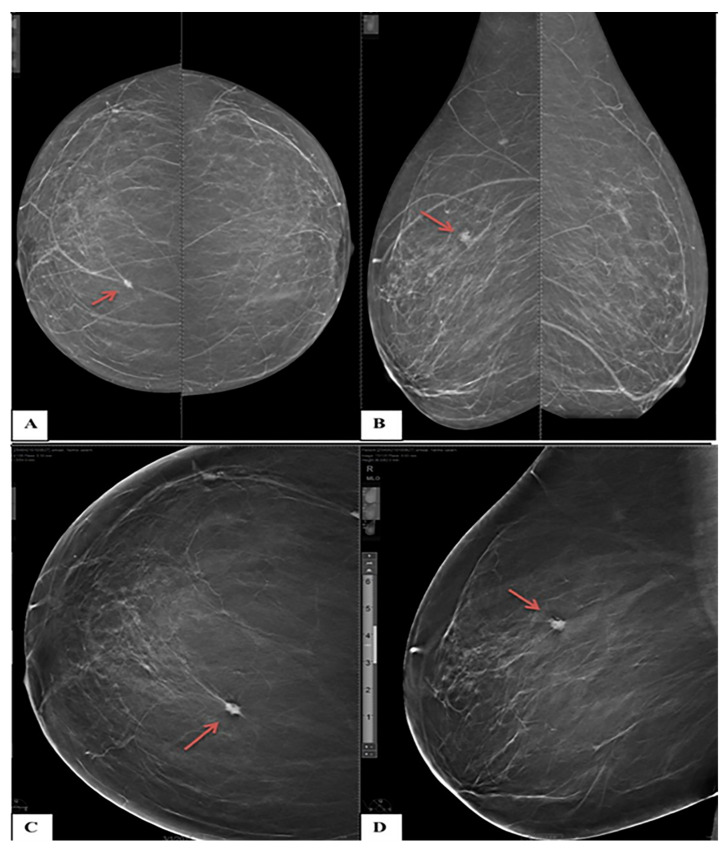
A 61-year-old female presented with right breast pain (**A**) Craniocaudal and (**B**) Mediolateral oblique DM images for both breasts show bilateral fatty breasts (ACR A). The upper inner quadrant of the right breast shows a small, oval, and ill-defined mass lesion (arrows). No microcalcifications (**C**) Craniocaudal and (**D**) Mediolateral oblique DBT images of the right breast show speculated margins of the right breast lesion (arrows). The lesion was classified as BI-RADS 3 based on DM and BI-RADS 4b based on DBT. Histopathological examination revealed invasive ductal carcinoma.

**Figure 6:**
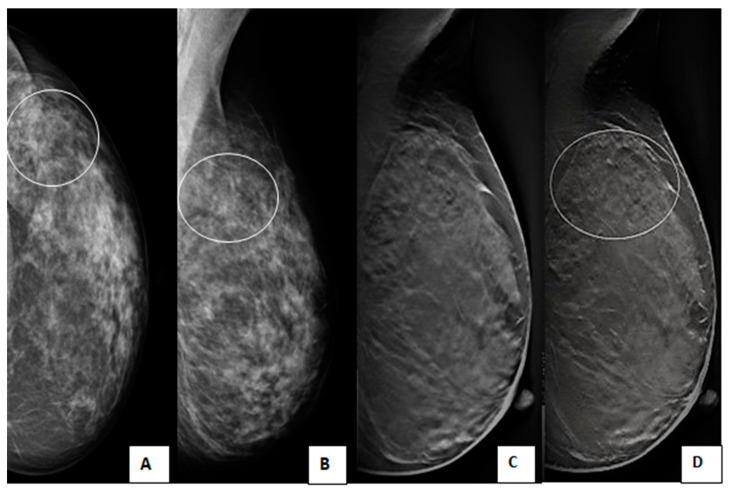
A 45-year-old female with a positive family history of breast cancer came for screening mammography. (**A**) Craniocaudal and (**B**) Mediolateral oblique DM images of the left breast show an upper outer quadrant speculated mass with obscured lower inner margin and surrounding architectural distortion (circles). (**C**,**D**) Mediolateral oblique DBT images of the left breast show an upper outer quadrant oval-shaped heterogeneous mass with circumscribed borders, but the lower inner margin is obscured (circles). The lesion was classified as BI-RADS 4a according to DM and downgraded to BI-RADS 3 according to DBT. Histopathological examination revealed invasive ductal carcinoma.

Figure 4, Figure 5 and Figure 6 show the sample cases from our study.

## 4. Discussion

Our study focused on equivocal BI-RADS classifications because the downgrading of BI-RADS 4 and 3 had crucial implications for case management. We pointed out the added value of DBT to the BI-RADS categorization. Most previous studies have focused on BI-RADS 3 category [21,22], and some have included category 0 [23]. In this study, we focused exclusively on BI-RADS categories 4, 3, and 0. Our study’s findings demonstrated that DBT has high diagnostic accuracy in the assessment of equivocal BI-RADS classes, as we discovered that integrated DM and DBT in BI-RADS resulted in a higher specificity (96.6%), sensitivity (98.7%), and accuracy (97.5%) for categorizing questionable breast lesions than DM or DBT alone. Our study’s findings demonstrated that DBT has high diagnostic accuracy in the assessment of equivocal BI-RADS classes, as we discovered that integrated DM and DBT in BI-RADS resulted in a higher specificity (96.6%), sensitivity (98.7%), and accuracy (97.5%) for categorizing questionable breast lesions than DM or DBT alone; sensitivity, specificity, and accuracy for the DM were 66.9%, 67.6%, and 67.3%, respectively, and 89.2%, 90.3%, and 90%, respectively, for DBT. In evaluating equivocal breast lesions, we discovered that DBT had much higher specificity, accuracy, and sensitivity than DM. These findings align with the results of many previous studies [15,17,23,24,25,26,27,28], which have identified that DBT enhances the specificity and sensitivity of DM. 

According to a study by Ali et al. [29], 3D DBT limited the need for additional mammographic views and repeated follow-up visits as it enhanced the depiction of all BI-RADS 3 lesions and lowered anxiety in women with suspicious lesions. They also concluded that scrolling 3D images for a specific view reduces the tissue overlap visible in 2D images, resulting in superior resolution and, consequently, greater diagnostic capabilities. Because in our research, better visibility of the lesions was achieved using further processing, such as zooming and adjusting contrast and brightness while examining digital images on a workstation, no further views were used to assess the lesions to limit the radiation dose.

Concerning DM, DBT resulted in a substantial change in the BI-RADS categories in 66.8% of the lesions, with 32% upgraded and 34.8% downgraded. This is in line with a previous study by Raghu et al. [17], who found that the final assessment of BI-RADS changed after using DBT. 

A notable finding in our research is the capacity of DBT to detect a higher number of lesions than DM (10.6%), with the majority of these missed lesions falling into BI-RADS 2 (52.6%). Tissue overlap was the most common reason for the omission of a lesion on DM, especially in females with dense breast parenchyma and radiographically inconspicuous lesions. Conversely, DBT can overcome these two issues, allowing better imaging of the lesion. The BI-RADS categories between DM and DBT were significantly upgraded because of these missing lesions on DM, giving DBT a major diagnostic advantage over DM. This finding is consistent with earlier research [15,20,22], which found that DM is less accurate than DBT in detecting breast lesions. A comparable study by Mansour et al. [30] reported that breast lesions were more detectable on 3D DBT, which discovered 158 out of 166 analyzed lesions (95.2%) and skipped only eight lesions (4.8%). In comparison, DM skipped 38 lesions (22.9%). Furthermore, DBT performed better in lesion interpretation, identifying and correctly diagnosing 141 lesions (84.9%) compared to only 55 lesions (33.1%) for DM. Another finding in our study was that the number of BI-RADS 4 and 3 lesions was reduced when using DBT, which is one of the possible benefits of DBT, as certain lesions classified as BI-RADS 4 and 3 on DM were reduced to BI-RADS 2 and 1 or upgraded to BI-RADS 5 by DBT. Consequently, the number of follow-up visits and biopsy rates were reduced. These results match those recorded in previous studies [18,31].

When we examined the AUCs, we discovered that DBT was much better than DM in detecting breast lesions (0.883 vs. 0.619, *p* = 0.0001), and integrating DM and DBT was significantly better than DM or DBT alone (0.971, *p* = 0.0001). This was consistent with many previous studies [32,33] that demonstrated that the AUC of integrated DBT and DM was higher than that of DM alone. 

Despite having higher diagnostic accuracy than DM, DBT was unable to identify several breast lesions. In our study, 18 breast lesions were incorrectly diagnosed using DBT (eight false negatives and 10 false positives). Eight masses were classified as BI-RADS 3, indicating that they were likely benign on DBT but were confirmed to be malignant by histopathology. Compared to DM or DBT alone, the integrated DM and DBT considerably reduced incorrectly diagnosed lesions (three false positives and one false negative). Owing to the increased perception of a partially or completely smooth border, Nakashima et al. [34] discovered that certain cancers are mistakenly recognized as benign on DBT images. 

Accordingly, our findings indicate that BI-RADS could be used in conjunction with an integrated DM and DBT protocol to improve accuracy in the diagnosis of inconclusive breast lesions, allowing for better lesion management to minimize the need for unnecessary biopsy and dramatically reduce women’s irritability while waiting for biopsy outcomes. Based on our findings, we propose the following strategies to deal with breast lesions. First, because of its lower cost and wider availability, DM can be used as the initial imaging technique. If the BI-RAD category according to DM yields a definite diagnosis of malignancy or benign (i.e., BI-RADS 5, 2, or 1), no further DBT is required, and patients can proceed to management. However, if the diagnosis is inconclusive (BI-RADS 4, 3, or 0), at this point, DBT is used, and the BI-RADS category is derived by integrating DBT and DM data.

Our research had some drawbacks. First, we only examined equivocal breast lesions (BI-RADS 4, 3, and 0) and ignored other BI-RADS classes (BI-RADS 5, 2, and 1). Second, the DBT accuracy in each breast density category was not addressed. Third, inter-reviewer agreement was lacking. Fourth, the integrated DM and DBT protocols have a greater cost and an increased radiation dose. Therefore, we recommend using the combination procedure only for indeterminate lesions when the BI-RADS category is unclear, following DM alone.

## 5. Conclusions

In conclusion, DBT strongly influences the BI-RADS classification in recognizing and characterizing equivocal breast lesions (BI-RADS 4, 3, and 0) classified by DM.

## Figures and Tables

**Table 1:** Patients’ data.

Variable	Value
Age, years, mean S ± D (range)	44.2 ± 7.3 (30–71)
Family history	
Negative family history	74 (50)
1st degree relative	44 (29.7)
2nd degree relative	30 (20.2)
Site of lesions	
Both breasts	4 (2.7)
Right breast	85 (57.4)
Left breast	59 (39.9)
Clinical presentations	
Asymptomatic	42 (28.3)
Breast mass only	91 (61.4)
Breast mass and edema	7 (4.7)
Breast mass and retracted nipple	8 (5.4)
ACR BI-RADS density	
A	7 (4.7)
B	50 (33.8)
C	78 (52.7)
D	13 (8.8)
Final diagnosis of 178 lesions	
Benign	104 (58.4)
Fibrocystic changes	43 (41.3)
Fibroadenomas	32 (30.8)
Normal	13 (12.5)
Postoperative scar	5 (4.8)
Duct ectasia	4 (3.9)
Granulomatous mastitis	3 (2.9)
Abscess	2 (1.9)
Benign phylloides	2 (1.9)
Malignant	74 (41.6)
Invasive duct carcinoma	52 (70.3)
Invasive lobular carcinoma	13 (17.6)
Ductal carcinoma in situ	6 (8.1)
Mucinous carcinoma	3 (4)

Unless otherwise indicated, data are numbered with the percentage in parentheses. SD: standard deviation, ACR: American College of Radiology, BI-RADS: Breast Imaging Reporting and Data System.

**Table 2:** DBT and DM findings.

Findings	DM (*n* = 159)	DBT (*n* = 178)
Site of lesion		
Both breasts	7 (4.4)	8 (4.5)
Right breast	91 (57.2)	102 (57.3)
Left breast	61 (38.4)	68 (38.2)
Type of lesion		
Mass	74 (46.5)	140 (78.7)
Asymmetry	55 (34.6)	-
Architecture distortion	13 (8.2)	8 (4.5)
Clusters of microcalcification with no underlying mass	9 (5.7)	4 (2.2)
Dense breasts (BI-RADS 0)	8 (5)	-
Dilated ducts	-	5 (2.8)
Asymmetric densities	-	8 (4.5)
Overlapped glandular tissue	-	13 (7.3%)
Characters of mass		
Mass margin		
Obscured on mammography, but speculated on tomosynthesis	35 (47.3)	55 (39.3)
Ill-defined	28 (37.8)	22 (15.7)
Well-defined	11 (14.9)	63 (45)
Mass Shape		
Irregular	33 (44.6)	65 (46.4)
Round	22 (29.7)	46 (32.9)
Oval	10 (13.5)	19 (13.6)
Macrolobulated	9 (12.2)	10 (7.1)
ACR BI-RADS density		
A	8 (5)	8 (4.5)
B	53 (33.3)	55 (30.9)
C	85 (53.5)	95 (53.4)
D	13 (8.2)	20 (11.2)

The data are presented as numbers with percentages indicated in parentheses. DBT: digital breast tomosynthesis, DM: digital mammography, BI-RADS: Breast Imaging Reporting and Data System, ACR: American College of Radiology.

**Table 3:** BI-RADS classification of breast lesions by DBT and DM in relation to the final histopathological diagnosis.

	DM			DBT		
	Malignant	Benign	Total	Malignant	Benign	Total
Not Seen	7 (3.9)	11 (6.2)	18 (10.1)	0	0	0
BI-RADS 0	2 (1.1)	6 (3.4)	8 (4.5)	0	0	0
BI-RADS 1	0	0	0	0	13 (7.3)	13 (7.3)
BI-RADS 2	0	0	0	0	48 (27)	48 (27)
BI-RADS 3	15 (8.4)	54 (30.3)	69 (38.7)	8 (4.5)	33 (18.5)	41 (23)
BI-RADS 4a	33 (18.5)	33 (18.5)	66 (37.1)	8 (4.5)	3 (1.7)	11 (6.2)
BI-RADS 4b	6 (3.4)	0	6 (3.4)	10 (5.6)	0	10 (5.6)
BI-RADS 4c	11 (6.2)	0	11 (6.2)	17 (9.5)	7 (3.9)	24 (13.5)
BI-RADS 5	0	0	0	31 (17.4)	0	31 (17.4)
Total	74 (41.6)	104 (58.4)	178	74 (41.6)	104 (58.4)	178 (100)

The data are presented as numbers with percentages indicated in parentheses. DBT stands for digital breast tomosynthesis, DM stands for digital mammography, and BI-RADS stands for Breast Imaging Reporting and Data System.

**Table 4:** Alterations in individual lesion BI-RADS grading on account of DBT to DM.

DM	DBT								
	BI-RADS 0	BI-RADS 1	BI-RADS 2	BI-RADS 3	BI-RADS 4a	BI-RADS 4b	BI-RADS 4c	BI-RADS 5	Total
Not seen			10 (5.6)	2(1.1)		4 (2.2)	2 (1.1)	1(0.6)	19 (10.6)
BI-RADS 0				6 (3.4)			2 (1.1)		8 (4.5)
BI-RADS 1									0
BI-RADS 2									0
BI-RADS 3		13 (7.3)	20 (11.2)	22 (12.3)	2 (1.1)	6 (3.4)	5 (2.8)		68 (38.2)
BI-RADS 4a			18 (10.1)	11 (6.2)	9 (5.1)		6 (3.4)	22 (12.3)	66 (37.1)
BI-RADS 4b								6 (3.4)	6 (3.4)
BI-RADS 4c							9 (5.1)	2 (1.1)	11 (6.2)
BI-RADS 5									0
Total		13 (7.3)	48 (27)	41(23)	11 (6.2)	10 (5.6)	24 (13.5)	31(17.4)	178 (100)

Information is presented as numbers with percentages indicated in parentheses. The colors indicate whether DBT was upgraded (blue), downgraded (orange), or maintained the same grade (green) as DM. DBT: digital breast tomosynthesis; DM: digital mammography; BI-RADS: Breast Imaging Reporting and Data System.

**Table 5:** The diagnostic accuracy of BI-RADS with DM, BI-RADS with DBT, and BI-RADS with integrated DBT and DM for confident diagnosis of breast malignancy.

Criterion	DM		DBT		DM + DBT	
	%	95%CI	%	95%CI	%	95%CI
Accuracy	67.3		90		97.5	
Specificity	67.6	60.8–74	90.3	85.5–94	96.6	93.2–98.6
Sensitivity	66.9	58.7–74.4	89.2	83–93.7	98.7	95.2–99.8
Positive likelihood ratio	2.1	1.7–2.6	9.23	6.1–14.1	29.2	14.1–60.4
Negative likelihood ratio	0.49	0.38–0.36	0.12	0.08–0.19	0.01	0–0.06
PPV	59.6	51.8–67.2	86.8	80.4–91.8	95.4	90.8–98.1
NPV	74.1	67.2–80.2	92.1	87.5–95	99	96.5–99.9

BI-RADS: Breast Imaging Reporting and Data System; AUC: area under curve; PPV: positive predictive value; NPV: negative predictive value; CI: confidence interval.

## Data Availability

The datasets used and/or analyzed during the current study are available from the corresponding author upon reasonable request.
